# Fish Intake during Pregnancy and Foetal Neurodevelopment—A Systematic Review of the Evidence

**DOI:** 10.3390/nu7032001

**Published:** 2015-03-18

**Authors:** Phoebe Starling, Karen Charlton, Anne T. McMahon, Catherine Lucas

**Affiliations:** School of Medicine, University of Wollongong, NSW 2522, Australia; E-Mails: pd266@uow.edu.au (P.S.); amcmahon@uow.edu.au (A.T.M.); cjl623@uowmail.edu.au (C.L.)

**Keywords:** pregnancy, fish, neurodevelopment, cognition

## Abstract

Fish is a source of several nutrients that are important for healthy foetal development. Guidelines from Australia, Europe and the USA encourage fish consumption during pregnancy. The potential for contamination by heavy metals, as well as risk of listeriosis requires careful consideration of the shaping of dietary messages related to fish intake during pregnancy. This review critically evaluates literature on fish intake in pregnant women, with a focus on the association between neurodevelopmental outcomes in the offspring and maternal fish intake during pregnancy. Peer-reviewed journal articles published between January 2000 and March 2014 were included. Eligible studies included those of healthy pregnant women who had experienced full term births and those that had measured fish or seafood intake and assessed neurodevelopmental outcomes in offspring. Medline, Scopus, Web of Science, ScienceDirect and the Cochrane Library were searched using the search terms: pregnant, neurodevelopment, cognition, fish and seafood. Of 279 papers sourced, eight were included in the final review. Due to heterogeneity in methodology and measured outcomes, a qualitative comparison of study findings was conducted. This review indicates that the benefits of diets providing moderate amounts of fish during pregnancy outweigh potential detrimental effects in regards to offspring neurodevelopment. It is important that the type of fish consumed is low in mercury.

## 1. Introduction

Fish is a source of several nutrients that are important during pregnancy for healthy foetal development including iodine, long chain omega-3 polyunsaturated fatty acids (LC*n-*3PUFAs), and vitamins A, D and B12 [1]. Guidelines from Australia [2], Europe [3] and the USA [4] encourage the consumption of fish during pregnancy. Recent studies indicate that pregnant women lack sufficient knowledge regarding the importance of iodine and LC*n*-3 PUFAs [5,6], nutrients that are present in fish and seafood. In addition, it appears Australian women are falling short of LC*n-*3PUFA intake recommendations during pregnancy [6,7]. On average, Australian women are consuming 33 g of fish per day and pregnant women an average of 28 g of fish per day, below Food Standards Australia New Zealand (FSANZ) recommended intakes [8]. As well as being a source of essential nutrients, fish are also a potential source of contaminants including mercury, polychlorinated biphenyls and dioxins [9]. Guidelines emphasising the health risks of methyl-mercury, with little mention of important nutrients found in fish, may be contributing to women consuming less than the recommended fish servings during pregnancy [10]. Thus the risks and benefits resulting from fish consumption need to be considered and scientific evidence should direct advice given to pregnant women to help them make the safest choice.

There are many documented health benefits from fish consumption with regard to foetal health, including improved neurodevelopment, increased birth weight and a reduced risk of spontaneous abortion [11,12]. This review focuses on neurodevelopmental outcomes for the foetus as much of the published research into fish consumption during pregnancy has focused on methyl-mercury, LC*n*-3PUFAs and iodine, all known to impact foetal neurodevelopment [13,14]. The aim of this review was to critically appraise literature investigating fish intake in pregnant women to assess the hypothesis that fish consumption during pregnancy positively influences foetal neurodevelopment. This review concludes with a discussion highlighting some of the methodological issues in researching associations between diet and infant neurodevelopment.

## 2. Experimental Section

### 2.1. Eligibility Criteria

Articles published in peer-reviewed journals between January 2000 and March 2014 were included in this review. Eligible studies were those with healthy pregnant women, full term births and offspring with no anomalies or diseases. Articles that investigated the relationship between the maternal consumption of fish or seafood and neurodevelopmental outcomes in offspring were included. Animal studies, studies not reported in English, and studies of populations exposed to contaminants were excluded. Articles directed at identifying suitable models to explain the relationship between components in fish or developing tools to analyse the risk-benefit of fish consumption without measuring neurodevelopmental outcomes were not deemed eligible for this review.

### 2.2. Search Strategy

Medline, Scopus, Web of Science, ScienceDirect and the Cochrane Library were searched using terms outlined in Table 1.

**Table 1:** Database search strategy.

	Search terms	Keywords searched	BOOLEAN operator
Term 1	Pregnant or pregnancy	Pregnan*	AND
Term 2	Fish or seafood	Fish seafood	OR AND
Term 3	Neurodevelopment or neurodevelopmental or cognition or cognitive	Neurodevelopment* cogniti*	OR

The Dietitians Association of Australia Process Manual for review of the Australian Dietary Guidelines [15] was the basis for reviewing the articles and guided the concluding evidence statement. The quality rating of the studies eligible for review was assessed based on the NHMRC guidelines for review of scientific literature [16].

## 3. Results

**Figure 1:**
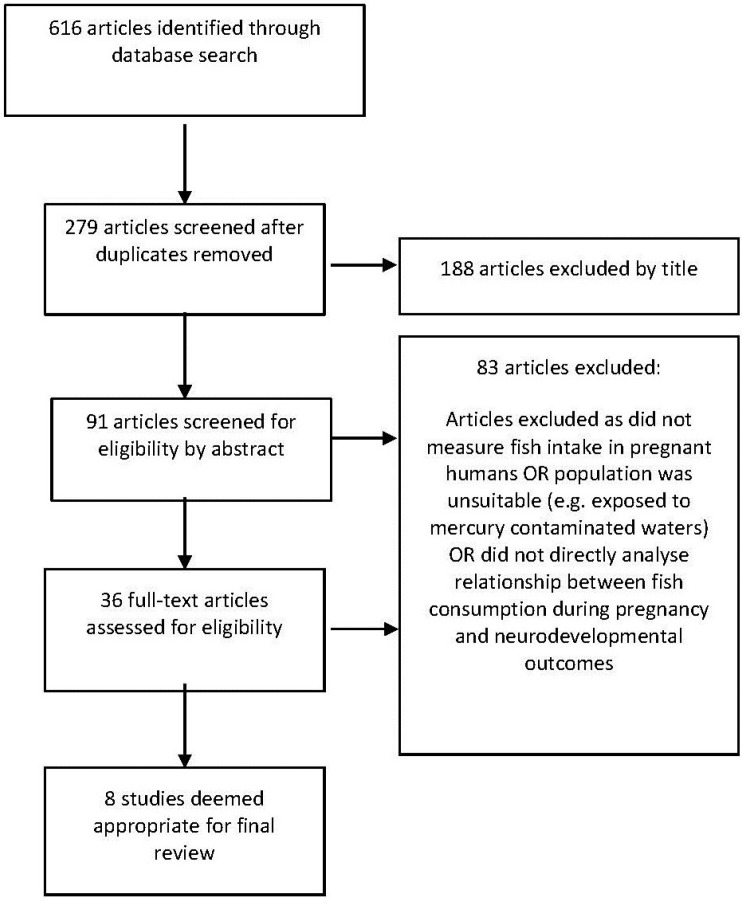
Flow diagram for inclusion of journal articles [17].

The initial search identified 279 articles after duplicates were removed, eight of which were suitable for inclusion. The PRISMA statement process was followed [17] as shown in Figure 1.

The search strategy did not yield any randomised controlled trials of fish intake in pregnant women and associated foetal neurodevelopmental outcomes. All eight studies included in the final review were observational in design. A summary of these articles and their quality rating is presented in Table 2.

**Table 2:** Summary and quality rating for reviewed articles.

Reference	Daniels *et al.*, 2004 [18]	Oken *et al.*, 2005 [19]	Hibbeln *et al.*, 2007 [20]	Gale *et al.*, 2008 [21]
Type of study	Observational cohort (Avon Longitudinal Study of Parents and Children-ALSPAC)	Prospective cohort (Project Viva)	Observational cohort (ALSPAC)	Observational cohort
NHMRC Level of evidence [20]	Level III-3	Level III-3	Level III-3	Level III-3
Population	Pregnant women living in Bristol & surrounds, United Kingdom (UK).	Pregnant women recruited in Massachusetts, United States of America (USA)	Pregnant women in Bristol & surrounds, UK.	Pregnant women recruited in Southampton, UK.
*N*—sample size	7421 mother-child pairs.	135 mother-child pairs	5449 children assessed.	217 mother-child pairs.
Method	Measured fish intake during at 32 weeks gestation by Food Frequency Questionnaire (FFQ) during. Breastfeeding, child fish consumption, maternal dental & lifestyle questionnaires. Neurodevelopmental testing of child completed by mother using ALSPAC adaption of the MacArthur Communicative Development Inventory (MCDI) at 15 months and Denver Developmental Screening Test (DDST) at 18 months of age.	Fish and seafood intake measured via a validated FFQ (calibrated for LC*n*-3PUFAs in blood) at 28 weeks gestation. Infant cognition measured using Visual Recognition Memory (VRM) paradigm at 6 months of age.	Seafood consumption assessed at 32 weeks gestation via FFQ. Postal questionnaires on diet, education, social, behavioural and developmental outcomes at child age: 6, 18, 30, 42, and 81 months. Wechsler Intelligence Scale for Children III used to assess Intelligence Quotient (IQ) at 8 years and Strengths and Difficulties Questionnaire (SDQ) conducted. Presented as percentage of children in the lowest quartile for WISC-III and ALSPAC development test subscales or in the suboptimum range of behavioural scores for the SDQ. Tested at 42 months, 7 and 8 years.	Two FFQs during pregnancy (at 15 weeks and 32 weeks) were used to estimate fish intake in early and late pregnancy. Cognitive & behavioural outcomes in offspring at 9 years using the Wechsler Abbreviated Scale of Intelligence. The SDQ was used to measure maladaptive behaviour.
Intervention/comparator	Fish intake: rarely/never, once a fortnight, 1–3 times per week, 4 or more times per week. Assumed each fish serve was 4.5 ounces (~0 g, 64 g, 255 g and 510 g per week).	Second trimester fish servings: more than 2 weekly fish servings compared to 2 or less. Did not convert servings to grams.	Comparing no seafood intake and 1–340 g per week with more than 340 g per week (3 servings is estimated as 340 g).	Fish servings per week in early and late pregnancy: never, less than 1, 1–2 times, 3 or more times. Oily fish servings in both early and late pregnancy: never, less than 1, 1 or more. Amount not specified in grams
Outcome	Fish consumption during pregnancy resulted in modest but significant improvement in developmental scores for language & social activity at 15–18 months age. Odds ratio (OR) and 95% confidence interval (CI) for high test score for MCDI: Vocabulary comprehension = 1.5 (1.1–2.0) for one or more serves compared to no serves. Social activity = 1.6 (1.2–2.2) for 1/fortnight, 1.7 (1.3–2.2) for 1–3/week and 1.8 (1.4–2.4) for 4+ serves/week compared to no serves.	Non-significant increase in VRM of 2.8 points for each additional weekly fish serving (95% CI = 0.2 to 5.4). When mercury confounder was adjusted for, this association became significant: 4.0 (1.3 to 6.7). Mothers consuming greater than 2 fish serves per week had infants with the greatest VRM scores.	Seafood intake during pregnancy was associated with a significant reduction in percentage of children with suboptimal IQ and behaviour test scores in 9 of 23 outcomes. Non-seafood consumers during pregnancy had children who scored lower on tests of verbal IQ at 8 years: OR (CI) for no seafood = 1.48 (1.16–1.90); some seafood 1.09 (0.92–1.20) compared with >340 g per week [overall trend: *p* = 0.004].	Oily fish consumption more than once per week versus no oily fish reduced the risk of hyperactivity. No association with fish consumption in early pregnancy and full scale IQ, however, total fish intake in late pregnancy of 1 to 2 serves per week was associated with having a child with higher IQ at age 9 years. Higher intakes (3 or more serves per week) did not show a statistically significant improvement. Regression coefficients (95% CI) for fish consumption and full scale IQ: less than once per week *vs.* no fish = 7.76 (0.38 to 15.1), once or twice per week vs. no fish = 6.91 (0.19 to 13.6). Verbal IQ & fish consumption: Increase of 7.32 (0.26 to 14.4) with fish consumption once or twice per week. 8.07 (0.28 to 15.9) with three or more serves per week.
Quality	Neutral	Positive	Neutral	Positive
Type of study	Prospective population-based cohort	Prospective cohort (Project Viva)	Prospective birth cohort	Birth cohort study
NHMRC Level of evidence [20]	Level III-3	Level III-3	Level III-3	Level III-3
Population	Pregnant women recruited throughout Denmark.	Pregnant women recruited in Massachusetts, USA.	Pregnant women living in Menorca, Spain.	Pregnant women recruited in Japan.
*N*—sample size	25,446 mothers-child pairs.	341 mother-child pairs.	392 mother-child pairs	498 mother-infant pairs
Method	Validated FFQ conducted at 25 weeks gestation to estimate fish intake. Standardised interview with mother used to assess child neurodevelopment at 6 and 18 months. Measured the odds of improved development scores due to fish intake. No individual comparison for each category—pooled estimate only.	Fish intake during pregnancy estimated via semi-quantitative FFQ. Peabody Picture Vocabulary Test (PPVT) and Wide Range Assessment of Visual Motor Abilities (WRAVMA) tested at ~38 months age of child and analysed for association with fish intake.	FFQ of typical diet during pregnancy completed 3 months after delivery and fish and shellfish/squid intake estimated. Neurodevelopment (as well as diet and physical activity) assessed when child was 4 years of age using the McCarthy Scales of Children’s Abilities (MCSA) tests—global cognitive scale & 5 subscales (perceptive-performance, memory, verbal, quantitative and motor).	Fish intake measured via FFQ 4 days after birth of child. Trained examiners conducted Neurodevelopmental testing of child was completed via a Neonatal Behavioural Assessment 3 days post birth (28 behavioural & 18 reflex items).
Comparator and Comparison	Fish intake in quintiles Weekly servings Categories: no fish (0 g), 1–2 servings (1–340 g per week), or 3 or more servings per week (over 340 g).	Fish consumption: No fish serves, less than or equal to 2 servings per week, greater than 2 servings per week.	Maternal fish intake of more than 2–3 times per week compared to up to once per week. No mention of intake in grams.	Maternal seafood intake in grams (average intake = 300–360 g per week).
Outcome	Highest 3 quintiles of fish intake resulted in improved motor, social/cognitive and total development scores at 18 months: OR (95%CI) = 1.28 (1.20, 1.38) for highest versus lowest quintile. This association was less obvious at 6 months (only the highest quintile showed significant improvement).	Offspring of women who ate fish more than twice a week scored significantly higher on WRAVMA drawing and total scores compared with no serves. OR (95%CI) for WRAVMA drawing = 6.0 (1.8, 10.2) for more than two serves per week compared with no serves. WRAVMA total score = 5.3 (0.9, 9.6) for more than two serves per week compared to no serves.	Pregnant women fish consumption greater than 2–3 times per week had children with significantly higher cognition and motor development scores compared to women consuming fish less than once a week. This association was only significant in children breastfed for up to 6 months. Greater than 3 serves per week was not associated with improved outcomes.	Seafood intake weakly (*p* = 0.1) correlated with motor development. Other measures of neurodevelopment not significant in either direction.
Quality	Neutral	Positive	Positive	Neutral

Studies were heterogeneous in methodology in regard to the covariates, neurological assessment tools, length of follow up statistical analyses. In addition, the type and amounts of fish consumed differed across study locations. Due to this heterogeneity a qualitative rather than quantitative approach was deemed appropriate for comparison and presentation of findings.

A study by Oken *et al.* tested children at six months of age using Visual Recognition Memory (VRM) paradigm and found a significant improvement of 2.8 points for each additional serving of fish (85–140 g) consumed by the mother during pregnancy [19]. Mendez *et al*. found that the length of breastfeeding influenced whether a significant difference was found in offspring neurodevelopment when comparing high and low fish intakes during pregnancy [24]. Authors reported that fish consumption two to three times per week during pregnancy was beneficial for children who were breastfed for less than six months. However, no statistical improvement was indicated for those children who were breastfed for longer than six months [24]. Gale *et al.* demonstrated no adverse effects on offspring neurodevelopment with maternal fish consumption during pregnancy equal to or greater than once per week. This study reported an improved verbal Intelligence Quotient (IQ) in offspring aged nine years in children born to mothers who consumed up to two servings of fish per week compared with children born to mothers who had not consumed any fish during late pregnancy (32 weeks gestation). This association was not significant for fish consumption in early pregnancy (15 weeks gestation) suggesting that fish consumption may be of more benefit during the third trimester [21].

A report from the Avon Longitudinal Study of Parents and Children (ALSPAC) found that fish consumption during pregnancy of one to three servings per week was shown to provide a modest but significant improvement in developmental scores of the offspring for language and social activity at fifteen to eighteen months of age [18]. A longer follow up of the ALSPAC cohort demonstrated a reduction in the percentage of children with suboptimal IQ at eight years of age amongst mothers with a high seafood intake (greater than 340 g) during pregnancy [20].

A smaller (*n* = 498) Japanese study did not demonstrate a positive or negative association between maternal fish intake during pregnancy and neurodevelopment as measured by the Neonatal Behavioural Assessment tool in infants at three days of age [25]. Conversely, results from a US cohort study demonstrated a significant improvement in IQ with consumption of more than two maternal servings of fish intake per week as assessed via ‘milestone’ achievement in children aged six months and eighteen months [23]. Results from a large Danish national birth cohort (*n* = 25,446) indicated a significant improvement in motor, cognitive and total developmental scores for eighteen month old children who were born to women within the highest three quintiles of fish intake during pregnancy. At six months, this improvement was only significant for children of women in the highest quintile of fish consumption, suggesting that age of testing may be relevant [22].

The overall quality rating score for the reviewed studies are presented in Table 2. The eight eligible studies were graded as negative, neutral or positive overall based on NHMRC guidelines for scientific literature reviews [16]. All studies were found to be either positive or neutral in regards to quality rating. Seven of the eight reviewed articles showed a beneficial impact on certain measures of offspring neurodevelopment with fish intake ranging from less than one to three or more servings of fish per week. Thus, the evidence in these cohort studies supports the current recommendations for fish consumption during pregnancy [2,3,4]. The level of this evidence is Grade C as per the ratings outlined in Table 3, adapted from Williams *et al.* [15] indicating that the body of evidence is supportive of fish intake during pregnancy.

**Table 3:** Evidence Rating Table [13].

Component	Rating	Comments
Evidence Base	Satisfactory	NHMRC Level III (cohort studies) with moderate risk of bias [20].
Consistency	Good	Seven out of eight studies demonstrated a positive association between fish intake and foetal neurodevelopment.
Clinical impact	Satisfactory	Trend towards improved neurodevelopment with significant results in several domains.
Generalisability	Good	All studies in pregnant women.
Applicability	Poor	A variety of populations studied from different countries where type of fish and the level of contaminants would likely vary.

## 4. Discussion

This systematic review of observational cohort studies demonstrates an association between consumption of one or more servings of fish per week during pregnancy and better offspring neurodevelopment outcomes. Suzuki *et al.* [21] was the only study to report a neutral effect of seafood intake on all neurodevelopmental outcomes. However, in that study neurodevelopment was assessed when the infant was only three days old. The seven studies, which demonstrated a benefit in neurodevelopment, had a follow up time ranging from six months to nine years. Thus it is possible that longer follow up may be needed to determine significant associations. This concept is supported by Oken *et al.* which found an improvement in neurodevelopmental scores at six months in only the highest quintile of fish intake while this improvement was evident in the highest three quintiles at eighteen months [22].

Mendez *et al.* [24] found that seafood consumption that excluded fish intake had a detrimental effect on neurodevelopment, while fish intake alone led to improved outcomes. Thus it is possible that the study of Suzuki *et al.* [25] may have detected a benefit had fish been considered separately from total seafood. Gale *et al.* [21] reported differences in outcomes associated with oily fish intake and total fish intake in early compared to late pregnancy. This suggests that the type of fish and timing of consumption during pregnancy may impact on neurodevelopmental outcomes of offspring. However, this information is limited to a single study, which was conducted in a relatively small sample (*n* = 217). More research in this area is required to draw sound conclusions.

It is important to note the number methodological limitations in research on diet and infant neurodevelopment that are present in these studies. This prevents the conclusion of a definitive relationship without further research, preferably clinical randomised controlled trials, and a proper meta-analysis.

Measuring dietary intake in cohort studies is problematic due to the difficulty in obtaining detailed information without causing significant subject burden. All identified observational cohort studies in the current review used food frequency questionnaires (FFQs) to assess fish intake. No studies reported adjusting results from the FFQ for energy intake, a recommendation made by Freedman *et al*. 2011 [26] to prevent attenuation. Three studies [19,21,24] reported on the frequency of consumption without specifying the weight of fish servings while the remaining studies made assumptions based on standard serving sizes, as to the quantity of fish consumed at each occasion. This limits the accuracy of a quantifiable conclusion as due to individual variability in the perception of a ‘serving size’.

Assessing cognitive development differences in infancy and childhood is fraught with difficulties due to the nature of childhood development and the accurate measurement of such. Firstly, children develop in ‘spurts’ rather than in a continuous fashion, which means they may slip in and out of the ‘normal’ reference ranges, particularly in the earlier years [27,28]. To combat this, it has been suggested that testing occurs at more than one time point [27] and that testing should extend beyond the first two years, preferable to school aged children in order to detect more subtle differences [28]. Only 4 studies tested at multiple time points [18,21,22,24], four beyond two years [20,21,23,24], and only two looked at children of school age [20,21].

Secondly, there are multiple interrelated factors which impact on neurodevelopment, and not all confounders were accounted for in all analyses. Maternal intelligence, alcohol consumption, smoking and breastfeeding practices were included as covariates in all studies. However, factors including ethnicity, paternal intelligence, the home environment, drug use, dietary patterns, supplement use and maternal responsiveness were not always measured. The ALSPAC study reports by Daniels *et al.* [18] and Hibbeln *et al.* [20] included the home environment as a confounder, but not paternal IQ. Conversely, the two studies by Oken *et al.* adjusted for paternal IQ but not the home environment [22,23]. The remaining four studies did not correct for either paternal IQ or home environment. No studies adjusted for maternal responsiveness which has been shown to be related to developmental outcomes independent of sociodemographic factors [28]. An intake of fish may reflect a health conscious diet and thus the positive effects may not be directly attributed to the fish but rather to the diet as a whole. Oken *et al.* considered maternal diet by classifying women as following a “prudent” or “western” dietary pattern [23]. Hibbeln *et al.* [20] also adjusted for maternal diet, while Mendez *et al.* [24] adjusted for both maternal and child diets. Other studies did not effectively account for dietary intake and are thus at risk of bias. Oken *et al.* [22], Mendez *et al.* [24] and Hibbeln *et al.* [20] measured supplement usage during pregnancy. Hibbeln *et al.* [20] reported that only 1.7% of women consumed fish oil supplements not affecting outcomes, however, this study did not consider supplements other than fish oil. Only Mendez *et al.* [24] included supplement usage as a confounder.

There is no universal standard for which neurodevelopmental tests are most appropriate for use in children of varying ages and at what age meaningful differences in neurocognitive development can be detected [29]. Performance in assessments can be significantly altered if the participant is hungry, tired or fearful of being in a strange place or being tested [27]. The accuracy of the tests for the population depends on when the test was standardised and within what population. In particular the Denver test, utilised in the ALSPAC study [18] has been criticised for its low specificity and potentially outdated ‘norm’ as it was standardised in 1980 [30]. Research on the reliability of parental reports on child development is conflicting [31], Daniels *et al.* [18], Hibbeln *et al.* [20] and Oken *et al.* [22] used developmental testing carried out by the mother and thus results may not be as reliable as those reported by other studies, which used trained professionals.

Due to the risks associated with consuming fish and seafood during pregnancy related to food safety and heavy metal contamination, pregnant women may question the necessity of including these foods in their diets, when nutrition supplements are readily accessible in Western countries [32]. A systematic review of randomised control trials examining LC*n*-3PUFA supplementation during pregnancy found no clear association between supplement use and infant cognitive outcomes [33]. This may be attributable to the synergistic effects of food [34] and associated with fish being a source of other nutrients which are important for infant development such as iodine and vitamin D. Presently, there have been no randomised control trials examining neurocognitive outcomes associated with prenatal multivitamin use and infant neurodevelopment, and these are unlikely to occur given ethical implications of such [35]. Recent research has suggested that effects of methyl-mercury on infant brain development may be mediated by LC*n*-3PUFA [36]. Because it is possible to consume fish and seafood safely during pregnancy, through following recommendations to limit high mercury species, it is prudent to recommend that pregnant women consume these foods, rather than rely on supplementation, in order to maximise infant neurodevelopment outcomes.

## 5. Conclusions

This review assessed the hypothesis that fish intake during pregnancy improves offspring neurodevelopmental outcomes. A review of the available evidence indicates that intake of fish during pregnancy is associated with positive foetal neurodevelopmental outcomes, as supported by seven of eight articles reviewed, which showed a beneficial impact on foetal neurodevelopment with one or more servings of fish per week compared with no fish intake. Based on the results from these observational studies the current recommendation of two to three servings per week appears appropriate. Randomised clinical trials have been conducted using fish oil supplementation in pregnancy, but not with fish considered as a whole food. Existing evidence is currently insufficient to inform advice regarding fish intake during pregnancy. Further well designed studies are required to strengthen the evidence base regarding the type and quantity of maternal fish consumption during pregnancy and associated neurodevelopmental outcomes in the offspring, while considering the contribution of mercury from fish-containing diets.
